# An Extraordinary Wet Season and a Surge of Melioidosis: A Retrospective Case Series From a Tropical Emergency Department

**DOI:** 10.1111/1742-6723.70325

**Published:** 2026-08-05

**Authors:** Colin Banks, Amanda McConnell, Christopher S. Heather, Maisie Stephen, Kristen‐Jürgen Schroeder, Isaac Tredinnick, Shannon Wong, Vinay Gangathimmaiah

**Affiliations:** ^1^ Townsville University Hospital Townsville Queensland Australia; ^2^ James Cook University Townsville Queensland Australia; ^3^ Cairns Hospital Cairns Queensland Australia

**Keywords:** *Burkholderia pseudomallei*, emergency service, melioidosis, sepsis

## Abstract

**Objective:**

Townsville, North Queensland, experienced record rainfall in the 2024–2025 wet season with an associated surge in the number of patients with melioidosis. A large proportion presented to the Townsville University Hospital (TUH) emergency department (ED), presenting an opportunity to assess the surge from an ED perspective.

**Methods:**

This was a retrospective case series of all patients with melioidosis that presented to TUH ED between 1 November 2024 and 30 April 2025. Cases for the entire region were sourced from the Statewide Reference Laboratory, and then individually assessed to ascertain whether they first presented to the TUH‐ED.

**Results:**

There were 57 patients with confirmed melioidosis that presented to the TUH ED in the 2024–2025 wet season, 40 males and 17 females. Risk factors for melioidosis were present in 51 (88%), with diabetes being the most common (47%). ED blood cultures were taken in 54 patients and were positive in 36 (67%). All patients were admitted, with a median length of stay of 11 days; 12 (21%) patients had an intensive care admission, and hospital mortality was eight (14%). Pulmonary involvement was evident in 79% and prostatic involvement in 30% of males. Meropenem was administered in ED to 53% overall, and 80% of those with severe illness.

**Conclusions:**

The surge of patients with melioidosis demonstrated a wide spectrum of infection foci and severity of illness. In melioidosis endemic regions following significant rainfall, we recommend to culture widely, administer meropenem for severe infections and consider melioidosis as a cause for unusual presentations.

## 1 Introduction

**FIGURE 1:**
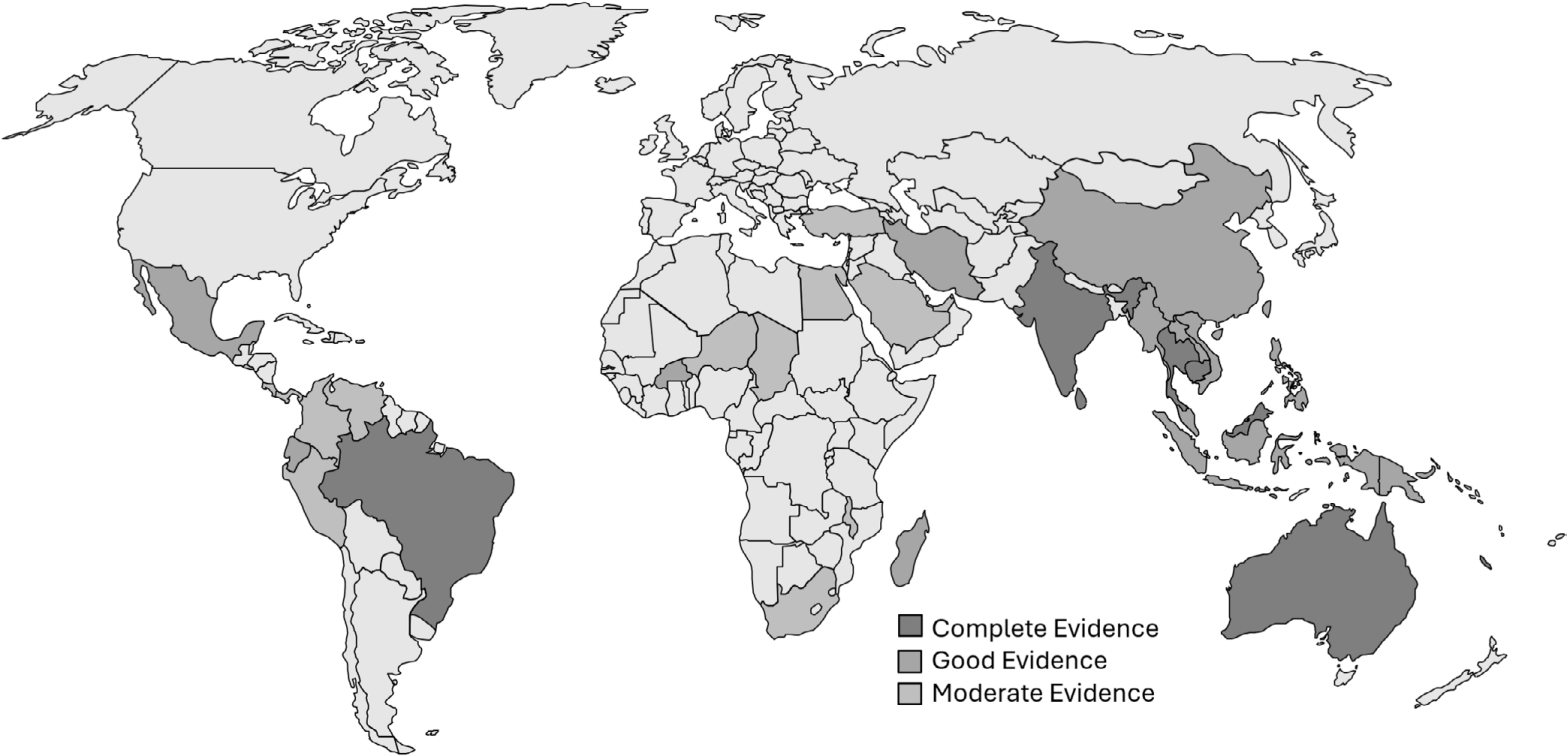
Global distribution of melioidosis. *Source:* Adapted from https://www.melioidosis.info/map.aspx.

Melioidosis is a potentially life‐threatening infection caused by *Burkholderia pseudomallei* , a gram‐negative bacterium that resides in the soil in tropical and subtropical regions around the world. In Australia, it predominantly occurs north of 20° latitude, although there have been sporadic cases further south [1]. See Figure 1 for a global depiction of where there is moderate or better evidence for melioidosis [2].

Serological studies in Thailand suggest the most common form of infection is asymptomatic seroconversion [3]. However, when clinical disease occurs, it can present with such a wide spectrum of manifestations that it has been described as a “great mimicker” [4]. Melioidosis typically presents as an acute illness, although chronic infection and reactivation of latent disease have also been described. Acute infection commonly presents as sepsis with pneumonia but can also present as pyogenic abscesses, cutaneous infections and septic arthritis. In Australia, prostatic involvement is commonly reported in males with melioidosis [5].

Melioidosis usually occurs in the wet season where there is a well‐documented association between rainfall extremes and melioidosis cases [6]. Infection may occur through transdermal inoculation, inhalation or ingestion of contaminated water or soil. The incubation period is 1–21 days [7]. Most patients who contract melioidosis have at least one of the following risk factors: diabetes, alcohol misuse, chronic lung disease, chronic kidney disease, immunosuppression or thalassaemia [4, 5]. It is usually diagnosed through culture (blood, urine, sputum or skin). There are serological tests available; however, these are difficult to interpret in endemic regions. Melioidosis treatment occurs in two phases: an initial intensive phase of intravenous induction therapy, usually meropenem or ceftazidime, followed by several months of eradication therapy with co‐trimoxazole. The exact duration is outlined in the Darwin Melioidosis Treatment Duration Guideline in Table 1 [8].

**TABLE 1:** The 2024 Darwin melioidosis treatment duration guideline.

Antibiotic duration—Determining focus	Minimum intensive phase (weeks)	Eradication phase (months)
Skin abscess	2	3
Bacteraemia with no focus	2	3
Pneumonia—Unilobar with no lymphadenopathy/ICU/+ve blood culture	2	3
Pneumonia—Multilobar with no lymphadenopathy/ICU/+ve blood cultures or Pneumonia—Unilobar with +ve blood culture but no lymphadenopathy or ICU	3	3
Pneumonia with lymphadenopathy or ICU or Pneumonia—Multilobar with +ve blood cultures	4	3
Deep‐seated collection and septic arthritis	4	3
Osteomyelitis	6	6
Central nervous system infection and mycotic aneurysms^a^	8	6

^a^ Lifelong suppressive therapy may be required following vascular prosthetic surgery.

Townsville is the largest tropical city in Australia with a population just over 200,000 [9]. The average annual rainfall is 1155 mm [10]. The 2024–2025 wet season (1 November 2024 to 30 April 2025) recorded 3035 mm of rain at Townsville airport—approximately three times the usual annual rainfall amount [11]. There were 91 cases of melioidosis within the Townsville region in 2025 which compares with an average of 25 annual cases for the 4 years prior [12]. These cases include patients referred in from rural hospitals and community settings. Within the emergency department (ED), there was a noticeable increase in the number of patients presenting with melioidosis providing an opportunity to study the caseload from the ED perspective. Of note, the ED has guidelines in place that recommend meropenem in the wet season for severe pneumonia and undifferentiated sepsis. The authors are not aware of any ED based case series of melioidosis despite the important role that EDs play with early recognition and treatment. The aim of this study was to describe the demographics, clinical presentation, treatment and outcomes of patients with confirmed melioidosis that presented to Townsville University Hospital (TUH) ED.

## 2 Methods

### 2.1 Design and Setting

This was a retrospective case series at a tertiary referral hospital in North Queensland providing care to 250,000 people [13]. In 2025, there were 96,855 patient presentations to TUH ED (personal e‐mail from Dr. Natalie Ly on 14 January 2026).

### 2.2 Population

Eligible patients presented to TUH ED between 1 November 2024 and 30 April 2025, and had culture‐confirmed melioidosis. Patients who were transferred from other healthcare facilities were excluded.

### 2.3 Data Sources

Melioidosis is a notifiable disease, and all cases are reported to the State Reference Laboratory located at TUH. Eligible patients were identified from a list of all cases within the region obtained from the State Reference Laboratory. Each case was interrogated through the integrated Electronic Medical Record (iEMR), with standardised data points entered into REDCap, then analysed in Microsoft Excel. Data were collected about demographic and clinical variables. The data extraction was done by four of the authors, who were given written instructions and to refer any queries to the lead author. There was no specific inter‐rater reliability process.

Daily rainfall was obtained from the Bureau of Meteorology.

### 2.4 Outcomes

Outcomes of interest were: Risk factors (diabetes, alcohol misuse, chronic lung disease, chronic kidney disease, immunosuppression or thalassaemia), presenting symptoms from the clinician's note, vital signs (presence of temperature > 38°C or systolic blood pressure < 90 mmHg) initial ED blood tests (full blood count, blood gas, C‐reactive protein (CRP) and blood cultures), culture specimen (e.g., blood, urine, sputum), location of infection (from discharge summary), imaging performed, antibiotic treatment in ED, organ support (vasopressors, high flow nasal cannula or invasive ventilation) during ED stay, intensive care unit (ICU) admission, ICU length of stay, surgical procedures requiring a general anaesthetic, hospital length of stay and in‐hospital mortality. Patients were designated as having severe illness in the ED if they were intubated, were treated with high flow nasal cannula, vasoactive medications or had a lactate greater than 4 mmol/L.

### 2.5 Sample Size and Data Analysis

A sample size calculation was not performed. All eligible cases were included. Data were analysed using Microsoft Excel. Categorical data were summarised using counts and proportions. Continuous data were summarised using medians and interquartile ranges (IQR).

### 2.6 Ethics Statement

This project was approved as a clinical audit by Townsville Hospital and Health Service Audit, Quality and Innovation Review (AQUIRE) Panel (THHSAQUIRE1890). No written consent has been obtained from the patients as there is no patient‐identifiable data included.

## 3 Results

There were 69 patients diagnosed with melioidosis in the Townsville referral region, 57 had presented through TUH ED and 12 were excluded as transfers. There were 40 males and 17 females. Median age was 62 years (IQR 50–71) with a range of 23–94 years. Of the 57, 13 (23%) identified as First Nations people.

**FIGURE 2:**
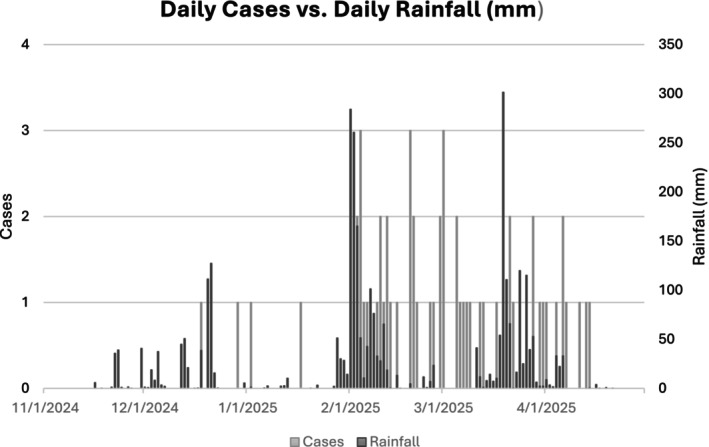
Daily melioidosis case presentations to Townsville University Hospital Emergency Department and rainfall at Townsville airport.

The daily caseload is plotted against the daily rainfall in Figure 2. Following the heavy rainfall at the start of February, there was a noticeable increase in the number of presentations with melioidosis.

Most patients, 51 out of 57 (88%), had at least one risk factor for melioidosis, with diabetes being the most common at 27 (47%). There were 18 (32%) patients with hazardous alcohol use, 15 (26%) patients that were immunosuppressed, 14 (25%) with chronic lung disease and nine (16%) with chronic kidney disease. Only six out of 57 (12%) patients had no risk factors.

Fever was the most common presenting symptom (39 out of 57, 69%), followed by 14 (25%) with dyspnoea and 12 (20%) with confusion. During the ED stay, 43 (75%) were febrile (≥ 38.0°C) and 12 (21%) were hypotensive (at least a single systolic blood pressure reading < 90 mmHg).

Of the samples taken in the ED, *B*. *pseudomallei* was cultured from blood in 36 (63%) patients, urine in 11 (19%), sputum in five (9%) and skin in one (2%) patient. In 15 patients (26%), *B. pseudomallei* was not cultured from ED specimens but was identified later from inpatient samples. Blood cultures were collected from 54 patients during the ED stay, yielding a positivity rate of 67%. Positive blood cultures took a median of 23 h to become positive (IQR 21–26 h, range 19–40 h). The three patients that did not get blood cultures done had presentations that did not suggest infection.

All 57 patients had white cell counts (WCC) reported. The median WCC was 13 × 10^9^/L (IQR 9–19, range 3–33). C‐reactive protein (CRP) was reported in 55 patients; the median CRP was 157 mg/L (IQR 74–241, range 1–455). There were 21 (37%) patients with CRPs greater than 200 mg/L.

The initial empiric therapy for suspected melioidosis is meropenem. In our case series, meropenem was administered in the ED to 30 of 57 patients (53%). There were 20 patients with severe illness as defined by any of the following:

- intubated (three patients),
- high‐flow oxygen (nine patients),
- vasoactive medications (eight patients) and
- lactate greater than 4 mmol/L (nine patients).

This group had positive blood cultures in 18 (90%) and a median CRP of 240 mg/L. In this cohort, 16 (80%) received meropenem in the ED.

All 57 patients were admitted to hospital. Median hospital length of stay was 11 days (IQR 7–24 days), with a range of 0.4 days (early death) to 144 days. There were 12 patients that were admitted to the ICU, with a median length of stay of 4 days (IQR 3–10 days). In total, 8 patients died before hospital discharge, corresponding to an in‐hospital mortality rate of 14%. All patients underwent computerised tomography (CT) scans, as it helps determine the duration of therapy. Most patients, 45 out of 57 (79%), had a final hospital discharge diagnosis of pneumonia. There was a great variety of pyogenic sources as presented in Table 2.

**TABLE 2:** Hospital discharge diagnosis for the 57 patients with culture‐confirmed melioidosis presenting to Townsville University Hospital Emergency Department during the 2024–2025 wet season. Multiple diagnoses possible for each patient.

Diagnosis	Number	Percentage
Pneumonia	45	79%
Prostate abscess	12	21%
Urinary tract infection	5	9%
Septic arthritis	4	7%
Osteomyelitis	3	5%
Skin abscess/ulcer	3	5%
Bacteraemia no source	2	4%
Mycotic abdominal aortic aneurysm	1	2%
Breast abscess	1	2%
Spleen abscess	1	2%
Perianal abscess	1	2%
Epididymo‐orchitis	1	2%

There were 16 patients who required a surgical procedure with a general anaesthetic. These procedures are outlined in Table 3.

**TABLE 3:** Surgical procedures requiring general anaesthesia in 57 patients with culture‐confirmed melioidosis presenting to Townsville University Hospital Emergency Department during the 2024–2025 wet season.

Procedure	Number
Abscess drainage‐prostate	6
Abscess drainage‐other	4
Joint washout	4
Laparotomy	2
Endovascular abdominal aortic aneurysm repair	1
Hip reduction	1
Heel debridement	1

## 4 Discussion

The melioidosis burden was particularly significant for the TUH ED during the 2024–2025 wet season, both in terms of severity of illness and methods of presentation. The surge in patient presentations compared to previous years was in keeping with the markedly increased rainfall and resultant flooding. Climate change is expected to increase the number of severe weather events. Such events have been linked to clusters of *B*. *pseudomallei* infection [1, 7].

There was a male predominance, with most patients in the middle decades of life. There were no paediatric cases. Risk factors in our cohort were similar to those reported previously [1, 6]. There were only six patients with no risk factors, none of whom died in hospital.

Imaging with CT was routinely performed in patients with melioidosis to identify additional foci of infection and determine duration of treatment. Pneumonia was the predominant clinical focus, identified in 79% of patients. There was prostate involvement in 30% of males, half of whom needed surgical drainage. This degree of prostate involvement has been previously reported in Australian literature [5, 14], but contrasts with Thailand where parotid abscesses are common and prostatic abscesses are rare [5].

Of the 57 cases, 54 had ED blood cultures taken, 36 (67%) of those were positive for *B pseudomallei* and 30 (53%) received treatment for melioidosis in the ED. This may be reasonable given that most patients were not critically unwell. Within the cohort of 20 with more severe illness, 16 (80%) received meropenem. Of the four severely unwell patients that did not receive meropenem in ED, the initial presentations were atypical for melioidosis and treatment was directed at the apparent primary problem: One patient presented with a ruptured abdominal aortic aneurysm (AAA). One presented with agitation and confusion and was later found to be septic. One presented with hyponatraemic seizures and was subsequently found to have pneumonia. The fourth presented predominantly with a urinary tract infection and a clear initial chest X‐ray (CXR), however, developed pneumonia which was evident on a CXR just 9 h later. It is worth acknowledging that the criteria used to designate severe illness is not a validated scoring system, and other scoring systems may have yielded different numbers.

There were 8 patients that died in hospital, giving a mortality rate of 14%. Two died within a few hours of presentation, both with markedly abnormal vital signs and both received meropenem. The remaining six died between 5 and 36 days (IQR 10–27 days).

This study has several limitations. The retrospective case series design could have resulted in selection and measurement biases. Selection bias due to missed eligible cases is unlikely as melioidosis is a notifiable disease and all cases were referred to the reference laboratory. Measurement bias due to reliance on clinician documentation in medical records may have impacted the accuracy of risk factors and presenting symptoms of melioidosis. The single centre design limits generalisability to other tropical emergency departments. Accessing data from the iEMR retrospectively is not real time, which limits any interpretation of clinical reasoning. Without a comparator period, it is not possible to comment on whether this cohort had different patterns or severity of illness. The presenting complaints were not correlated with the final discharge diagnosis.

The wet season of 2024–2025 was truly remarkable with about three times the usual rainfall and significant flooding throughout the city. During this period, the number of cases of melioidosis through the TUH ED alone was more than double the entire region's typical annual caseload.

There are three take home points for the ED clinician working in melioidosis endemic areas in the wet season. First, culture early and broadly. Take multiple cultures—not just blood. Second, if the patient is critically unwell, give meropenem early. Third, in patients with other infective syndromes with risk factors who are not responding to usual therapy, consider escalating empiric cover to include meropenem, particularly in the days to weeks after substantial rainfall and flooding.

This case series highlights the spectrum of illness that melioidosis can cause, both in location of infection and in severity of illness. Nearly all patients had at least one risk factor; inpatient mortality was significant, and the majority of patients had a final diagnosis that included pneumonia.

## Ethics Statement

This project was approved as a clinical audit by Townsville Hospital and Health Service Audit, Quality and Innovation Review (AQUIRE) Panel (THHSAQUIRE1890).

## Consent

The authors have nothing to report.

## Conflicts of Interest

The authors declare no conflicts of interest.

## Data Availability

The data that support the findings of this study are available from the corresponding author upon reasonable request.
